# Microbiota in foods from Inuit traditional hunting

**DOI:** 10.1371/journal.pone.0227819

**Published:** 2020-01-14

**Authors:** Aviaja L. Hauptmann, Petronela Paulová, Lars Hestbjerg Hansen, Thomas Sicheritz-Pontén, Gert Mulvad, Dennis S. Nielsen

**Affiliations:** 1 Greenland Center for Health Research, Ilisimatusarfik—University of Greenland, Nuuk, Greenland; 2 The Greenland Institute of Natural Resources, Nuuk, Greenland; 3 Department of Food Science, The University of Copenhagen, Frederiksberg, Denmark; 4 Institute of Experimental Endocrinology, Biomedical Research Center, Slovak Academy of Sciences, Bratislava, Slovakia; 5 Department of Environmental Science, Aarhus University, Roskilde, Denmark; 6 Centre of Excellence for Omics-Driven Computational Biodiscovery (COMBio), AIMST University, Kedah, Malaysia; 7 Natural History Museum of Denmark, University of Copenhagen, Copenhagen, Denmark; University of Maine, UNITED STATES

## Abstract

The foods we eat contain microorganisms that we ingest alongside the food. Industrialized food systems offer great advantages from a safety point of view, but have also been accused of depleting the diversity of the human microbiota with negative implications for human health. In contrast, artisanal traditional foods are potential sources of a diverse food microbiota. Traditional foods of the Greenlandic Inuit are comprised of animal-sourced foods prepared in the natural environment and are often consumed raw. These foods, some of which are on the verge of extinction, have not previously been microbiologically characterized. We mapped the microbiota of foods stemming from traditional Inuit land-based hunting activities. The foods included in the current study are dried muskox and caribou meat, caribou rumen and intestinal content as well as larval parasites from caribou hides, all traditional Inuit foods. This study shows that traditional drying methods are efficient for limiting microbial growth through desiccation. The results also show the rumen content of the caribou to be a highly diverse source of microbes with potential for degradation of plants. Finally, a number of parasites were shown to be included in the biodiversity of the assessed traditional foods. Taken together, the results map out a diverse source of ingested microbes and parasites that originate from the natural environment. These results have implications for understanding the nature-sourced traditional Inuit diet, which is in contrast to current day diet recommendations as well as modern industrialized food systems.

## Introduction

Culture-independent investigations of our foods have led to an increasing understanding that foods are ecosystems [1–4], and that these ecosystems are a source of microbes for our gastrointestinal tract [5,6]. Concurrently, the microbiota of traditional foods that are prepared by artisanal and non-standardized methods are stirring interest in science [7] and in popular scientific literature [8]. Non-urban traditional lifestyles have been associated with distinct gut microbiota relative to urbanized lifestyles. The gut microbiota of individuals from a spectrum of lifestyles of non-industrialized communities from hunter-gatherers to rural agricultural lifestyles have been described and associated with diet and lifestyle [9–11]. The pre-colonial traditional lifestyle of the Inuit of Greenland, *Kalaallit*, is in stark contrast to the majority of the world’s traditional lifestyles and does not match current day recommendations for a healthy diet [12]. Prior to colonization, the diet of the Greenlanders was almost exclusively animal-sourced and characterized by simple food preparation in the natural environment [13–15]. Succeeding colonization and the liberation of the trade of imported foods to the Inuit in Greenland in the 1860s, the Greenlandic diet has transitioned to a predominantly western diet [16–18]. Traditional foods prepared in the natural environment are still a culturally and nutritionally important part of the Inuit food culture, but knowledge and skills associated with these foods are gradually disappearing [16,19].

The extent of variation in the microbiota across foods, meals and diets is not well understood [5]. In this study, we assessed the biodiversity of dietary microbial input from traditional land-based hunting activities in Greenland. We assessed foodstuffs that have traditionally been eaten in connection to hunting activities focusing on foods that were consumed with no microbial elimination, namely dried meat of caribou and muskox, rumen content of caribou as well as larval parasites found in caribou hides in wintertime. Caribou intestines are also traditionally eaten. Although these are eaten boiled, cecum samples were also included in the study as the cecum is a potential source of a large number of microbes that may survive initial kill-steps. The process of caribou hunting and meat drying among Inuit has been described in great technical detail [20]. The fermented rumen-content of the caribou has been highlighted as a rich source of nutrients in the Inuit diet [14,21]. The intestines of the caribou are utilized as a good source of protein, fat and iron [21]. Fly larvae from the Oestridae family are found as parasites with a lifecycle step in caribou hides, from where the grubs have been collected and eaten by the Inuit during caribou hunting, though little is known about their nutritional potential [22]. Among the well-described Nunamiut Eskimos of Alaska an 80% dependence on caribou for their subsistence in more recent history has been reported [20]. Still today, hunting for caribou through the summer is an important activity, which ensures food for winter in Greenland. In Greenland, however, the diversity of foods from these activities have decreased to comprising mostly dried and fresh meat.

The microbial composition in the rumen of Norwegian and Russian populations of reindeer have been described previously [23–25]. Bacteroidetes and Firmicutes were shown to be dominant phylotypes in rumen of both reindeer fed a lichen-rich diet and reindeer fed a concentrate pellet-diet [23]. These studies have emphasized the rumen of the domesticated reindeer with connection to methane-emission [23,25] and lifestock health [24] and did not consider the importance of this source of microbes for human consumption. To the best of our knowledge, this is the first assessment of the microbial composition of traditionally dried caribou and muskox. We assessed the microbial composition and biodiversity of foods resulting from traditional hunting activities in Greenland by use of amplicon sequencing targeting the 16S rRNA gene with Illumina NexteraSeq technology. We investigated whether meat from different species of animals develop distinct microbial communities when dried and whether the traditional process of drying meat shares features with traditional meat curing by selecting for specific microbes. The aim of this study is to understand which factors influences the microbiota on non-industrial animal-sourced foods and to initiate a discussion of how traditional lifestyles among the Inuit might have played a role in shaping the microbiota of this population with its unusual diet.

## Materials and methods

### Sampling

**Fig 1:**
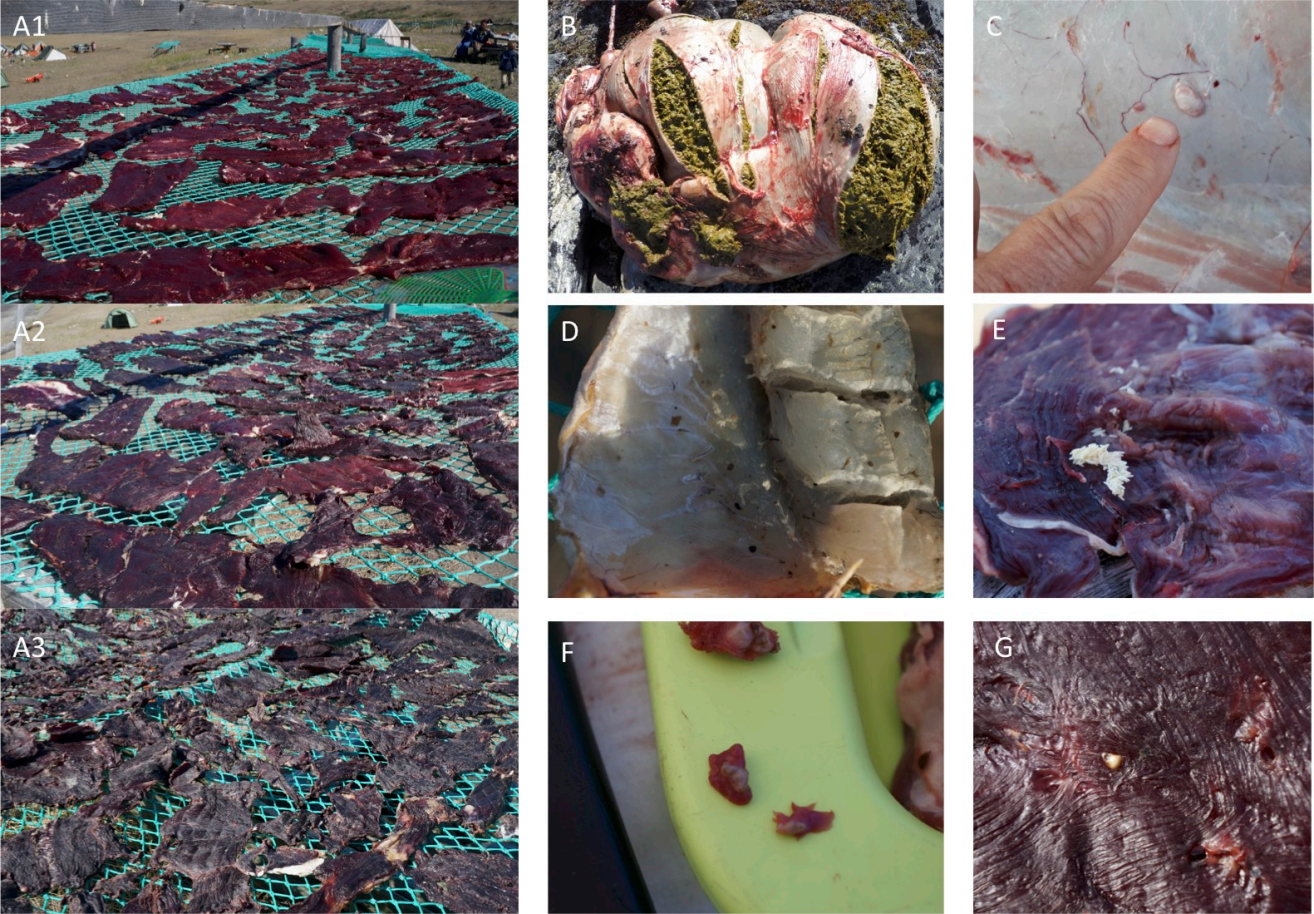
Photos. A1 drying meat day 0, A2 drying meat day 2, A3 drying meat day 3, B caribou rumen, C Oestridae larva inside caribou hide, D visible traces of blowfly excreta on drying fish (ogac), E blowfly maggots, F and G *Taenia* cf. *krabbei* in fresh and dried meat respectively.

The population of caribou (*Rangifer tarandus groenlandicus*) around Kangerlussuaq, Greenland (66.70°N, -51.44°W) assessed in the present study is an indigenous population estimated to be of about 58,900 individuals in 2016 [26]. This subspecies differs slightly from the feral Norwegian reindeer subspecies *R*. *t*. *tarandus*, which was imported to other regions of Greenland in 1952. The muskox (*Ovibos moschatus*) population assessed was introduced to the Kangerlussuaq area from Northeast Greenland during the 1960s after which they have been hunted along the caribou [27]. One caribou and two muskoxen were hunted at Angujaartorfik, Kangerlussuaq fjord, during the beginning of August 2017. All animals sampled for this study were killed as part of local hunting activities and were not killed for the purpose of research. All animals were hunted by licensed hunters according to local regulations overseen by the Greenland Ministry of Fisheries, Hunting and Agriculture. One member of the research team took part in hunting activities and was responsible for sampling of all the samples included in this study, but did not take part in the sacrifice of the animals included in the study. Two muskoxen were included to test for the potential difference in microbial composition on meat from different individuals. A simple sampling protocol was developed based on the need for preventing contamination of samples in the highly uncontrolled environment of the camping site. Camping meant limited access to cooling and the need for long term storage of samples with no access to cooling or lab facilities for at least a week. Caribou and muskox meat were cut up and laid to dry on nets at hunting camp in Angujaartorfik (Fig 1A). Caribou rumen (Fig 1B) and cecum were sampled instantly at the hunting-site of the caribou. The Angujaartorfik hunting camp is a natural area located away from any urban structures, which has been used for hunting by the Inuit for almost a millennium [28]. Microbes of drying meat were sampled by swabs once daily until the meat was assessed as being done drying. This assessment was carried out by local experts, who assess the progression of the drying process by feeling and bending the drying pieces of meat. In summary, all samples were taken using sterile cotton swabs (Aptaka, Canelli, Italy). The swapping area was approximately 100 cm^2^, which was ensured using a piece of laminated cardboard with a 10x10cm hole. The cardboard was not allowed to touch the sampled meat during sampling. To avoid re-sampling of previously sampled pieces of meat the sampling was initiated at one end of the drying rack and at each new sampling time pieces of meat were taken further down the drying rack. Edges of cotton swabs were put into 5 mL Eppendorf tubes® (Eppendorf, Hamburg, Germany) containing 3 mL RNA*later*^™^ (Invitrogen, Vilnius, Lithuania). Negative controls, 5 mL Eppendorf tube® containing 3 mL RNA*later*^™^, were brought on sampling trips and stored and transported alongside other samples. No DNA was detected in negative samples. At all sampling times for meat, samples were taken in triplicate from three individual pieces of meat. Some samples were discarded either because of being compromised during transportation (if Eppendorf tubes® had leaked during flight) or due to unsuccessful extraction of DNA. Microbiota from parasitic Oestridae larvae (*Hypoderma tarandi*) were sampled on October 29^th^ 2018 (Fig 1C). In short, a piece of caribou hide was cut off at hunting-site by a licensed hunter and kept frozen and transported to the lab. In the lab, three larvae were cut out from the skin using sterile scalpels. Using unused sterile scalpels, the larvae were removed from the hole in the skin. All three larvae were put into the same 5 mL Eppendorf tube® containing 3 mL RNA*later*^™^. The larvae are only present in caribou hides in winter time and winter hunting is only allowed by certified trophy hunters. Therefore, it was not possible to obtain samples alongside the other samples and consequently the larva microbiota was processed separately. Full list of final samples (*n* = 32) included in this study are listed in Table 1. Three fish (*Gadus ogac*) were also caught during the hunting trip. The fish were dried as meat described above. Ogac samples were included to test for the potentially different microbial compositions on meat from different animal species.

**Table 1:** Sample overview and metadata.

*Sample name*	*Chao1*	*Richness*	*Shannon e*	*Species*	*Fresh/Dry*	*Type*	*Drying* *days*	*Water* *activity*
*M1-0A*	112.5	52	2.47	MUSKOX	F	Meat	0	
*M1-1A*	40.6	39	2.11	MUSKOX	D	Meat	1	
*M1-1B*	50	50	2.17	MUSKOX	D	Meat	1	
*M1-1C*	187.5	43	2.06	MUSKOX	D	Meat	1	
*M1-2A*	66.5	62	2.59	MUSKOX	D	Meat	2	
*M1-2B*	91.5	87	3.24	MUSKOX	D	Meat	2	
*M1-3A*	86.5	80	2.87	MUSKOX	D (done)	Meat	3	0.39
*M1-3B*	189.2	184	3.85	MUSKOX	D (done)	Meat	3	
*M2-0A*	151.9	148	3.55	MUSKOX	F	Meat	0	
*M2-0B*	178.5	66	2.36	MUSKOX	F	Meat	0	
*M2-0C*	72.2	71	2.44	MUSKOX	F	Meat	0	
*M2-1A*	141.3	139	3.6	MUSKOX	D	Meat	1	
*M2-1B*	111.3	98	3.21	MUSKOX	D	Meat	1	
*M2-2A*	80.1	70	2.63	MUSKOX	D	Meat	2	
*M2-2B*	104.6	101	3.17	MUSKOX	D	Meat	2	
*M2-3A*	57.8	54	2.05	MUSKOX	D	Meat	3	
*M2-3B*	48.5	48	2.35	MUSKOX	D	Meat	3	
*M2-3C*	117.5	77	2.91	MUSKOX	D	Meat	3	
*M2-4A*	105.1	69	2.78	MUSKOX	D (done)	Meat	4	0.53
*M2-4B*	130.9	119	3.16	MUSKOX	D (done)	Meat	4	
*C-0A*	49.7	48	1.98	CARIBOU	F	Meat	0	
*C-0B*	25	25	1.27	CARIBOU	F	Meat	0	
*C-0C*	48	46	2.14	CARIBOU	F	Meat	0	
*C-1A*	102.9	98	2.85	CARIBOU	D	Meat	1	
*C-1B*	61.5	49	2.04	CARIBOU	D	Meat	1	
*C-2A*	75.4	72	2.79	CARIBOU	D (done)	Meat	2	0.6
*C-2B*	50.7	49	2.16	CARIBOU	D (done)	Meat	2	
*C-Gut*	27	27	2.82	CARIBOU	F	Cecum	0	
*C-Sto*	442.2	441	4.58	CARIBOU	F	Rumen	0	
*Larvae*	143	143	3.05	OESTRIDAE	F	Parasite	0	
*OA*	63.5	57	2.28	OGAC	D	Meat	4	0.77
*OB*	103.5	100	3.11	OGAC	D	Meat	4	

Sample names contain following information: Species (M: muskox, C: caribou, Larvae: Oestridae fly larvae, O: ogac), Individual (M1 and M2 are different muskox individuals), days of drying (from 0–4), and finally replicate number (A-C, some replicates are missing as explained in methods section).

Water activity of dried samples was measured using Decagon Pawkit Water Activity Meter (DECAGON, Pullman, Washington, USA) according to manufacturer’s manual.

### DNA extraction and sequencing

The bacterial compositions were determined using NextSeq-based (Illumina, CA, USA) high throughput amplicon sequencing targeting the V3 region of the 16S rRNA gene. The samples were transported cooled and were kept frozen after transportation. Following sample delivery, samples were thawed and vortexed. Precipitation of RNA*later*^™^ was observed in some tubes, which were then heated and vortexed according to manufacturer’s instructions (Thermo Fischer Scientific, MA, USA). DNA was isolated using the Bead-Beat Micro AX Gravity Isolation Kit (A&A Biotechnology, Poland) following the manufacturer’s instructions and the extracted DNA stored at -20°C until use. Sequencing libraries were constructed as described previously [29] and sequenced in a single NextSeq Illumina 2*150 bp run performed according to the manufacturer’s instructions. The presence of virulent *Shigella* was tested by PCR using primers specific for the *virA* gene, as previously described [30].

RNA extraction from samples was attempted but the limited amount of sample material did not allow for extraction of sufficient amount of RNA for analysis.

### Data analysis

Read pairs were merged using Usearch version 10.0.240 *fastq_mergepairs* [31]. Merged consensus sequences were quality checked using FastQC version 0.11.5 using default settings [32]. Usearch *fastq_filter* was used for quality filtering with minimum quality score of 25 and minimum length of 120 bases after which the sequences were quality checked with FastQC again. Quality filtered consensus sequences were pooled and pooled data was run through the Usearch pipeline for 97% identity OTU picking. In summary, unique sequences were identified with *fastx_uniques*, OTUs were clustered with *cluster_otus*, which also removes singletons and chimeras, finally the OTU table was created with *otutab* at default setting with a threshold of 97% identity. OTU table was normalized to 10,000 sequences/sample with *otutab_norm*. Prior to normalization the number of sequences ranged from a minimum of 75 to a maximum of 113,600 sequences and an average of 70,967 across the samples. Alpha diversity metrics were calculated with usearch *alpha_div* and taxonomy was assigned using usearch *sintax* with cutoff 0.7 and the RDP 16S database version 16 [33].

Beta diversity, specifically non-metric multidimensional scaling (NMDS and ANOSIM) was assessed in R version 3.5.0 [34]. NMDS employing Bray-Curtis distances was conducted using packages vegan version 2.5–1 [35] and labdsv version 1.8–0 [36].

## Results and discussion

### Traditional drying methods for caribou and muskox are efficient for limiting microbial growth

**Fig 2:**
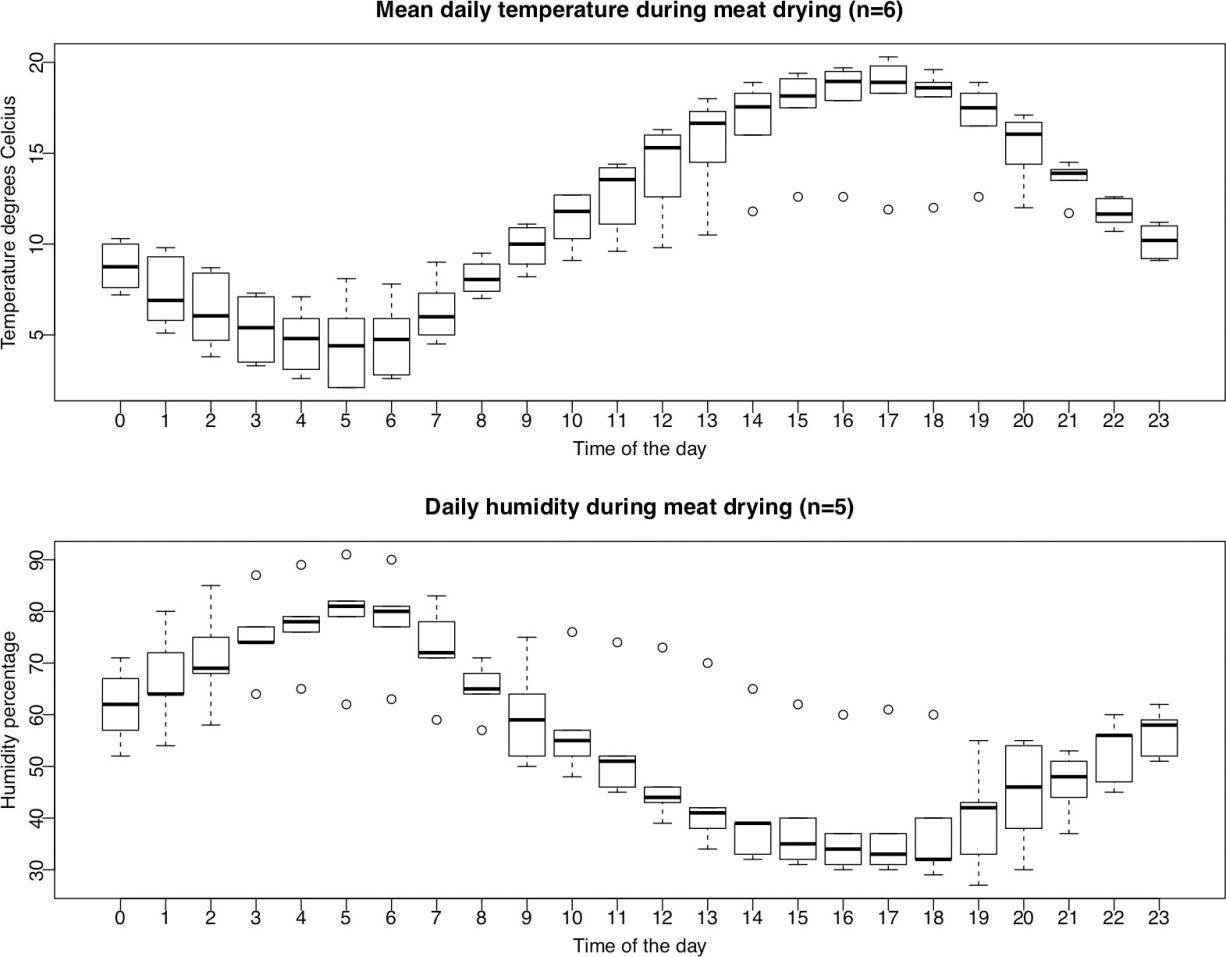
Temperature and humidity. Mean daily temperatures and humidity measured at nearby weather station Kangerlussuaq Airport between 1^st^ and 6^th^ of August (temperature) and 2^nd^ and 6^th^ of August (humidity, data for August 1^st^ not available). Source: Danish Meteorological Institute weather archive.

Angujaartorfik is characterized by continental climate and mean day temperatures measured at the nearest weather station of Kangerlussuaq Airport were up to above 20°C during days of the hunting and drying (Fig 2) [37]. The final water activity of dried meat was measured to be 0.39 for muskox individual 1, 0.53 for muskox individual 2 and 0.6 for caribou (Table 1, Fig 1). Based on considerations of microbial growth and spoilage Binford discussed the relationship between temperature and humidity for drying caribou among Inuit [20]. In case of high temperatures optimal for microbial growth and meat spoilage a high degree of desiccation is sought for, while at low temperatures cold-storage is more efficient [20]. The temperature and humidity during drying of sampled meat are shown in Fig 2. The processing of muskox and caribou at Angujaartorfik follows the logic described by Binford in which the warm and dry climate leads to the choice of drying for storage, thus adhering to traditional drying methods.

At water activities of 0.6 and below no microbial proliferation is expected [38]. This confirms that the evaluation of dryness by locals was effective for assessing when drying process was finalized. Based on the above considerations it can be concluded that drying of meat using traditional methods of the Inuit seems efficient in controlling microbial spoilage. It is important to note here that traditional food preparation in Greenland being an outdoor and seasonal activity is highly dependent on climate and that the climatic conditions available during the current study were optimal for desiccation. Unsolicited growth of mold can be observed on drying meat and was observed by authors on drying whale meat (a_w_ 0.85) in the area around Nuuk (64°N, -51°W) that has a colder and more humid coastal climate. Finally, it should be noted that today the dried meat is normally stored in freezers after drying to prevent spoilage. This means that while microbial growth is inhibited by drying the microbial community is still viable and can regain growth under favorable conditions with higher a_w_. Prolonged freezing might select for a more psychro-tolerant microbiota with a different composition than established for the samples of traditional foods described here. Additional research is encouraged to assess the impact of modern storage of these traditional foods such as prolonged freezing as well as complementary research assessing the active fraction of microbes on traditional Inuit foods.

### Microbial input from the environment determines the microbial composition of dried meat

**Fig 3:**
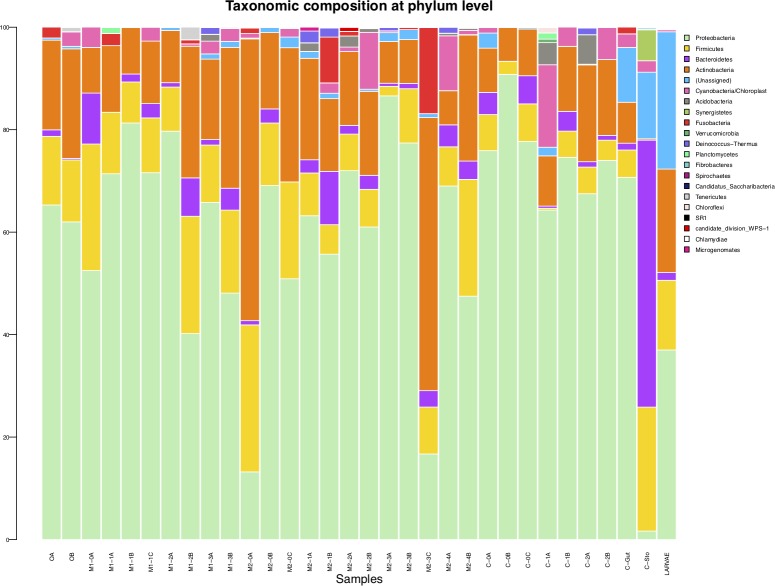
Taxonomic composition of all samples at phylum level. Please refer to Table 1 for overview of samples and explanation of sample labels.

**Fig 4:**
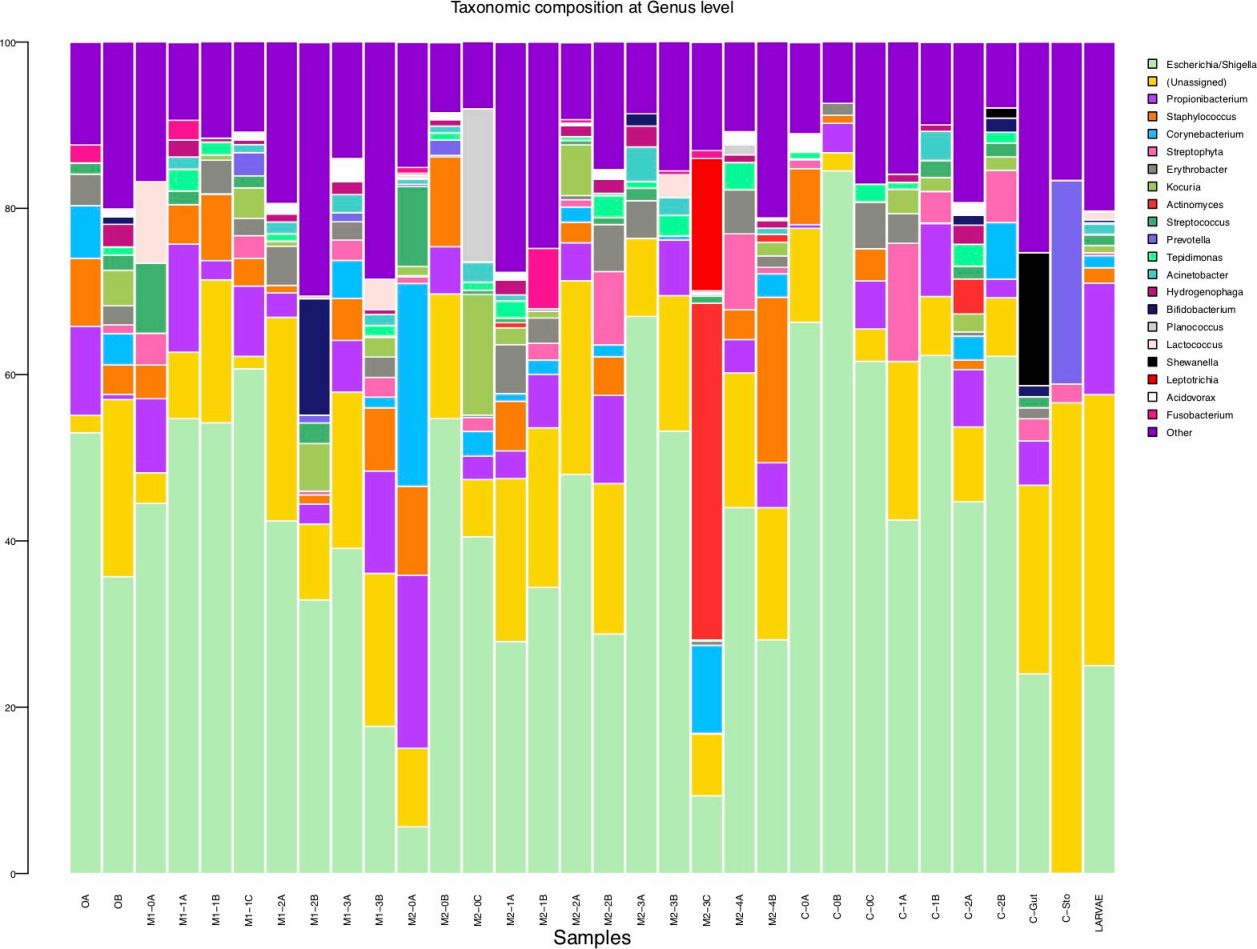
Taxonomic composition of all samples at genus level. The figure includes the top 20 genera across all samples. Please refer to Table 1 for overview of samples and explanation of sample labels.

Dried meat samples of muskox, caribou and fish (ogac) are generally dominated by the phylum Proteobacteria as well as Firmicutes and Actinobacteria among the 20 different observed phyla (Fig 3). Of other dominant phyla the samples show varying fractions of Bacteroidetes, Cyanobacteria/Chloroplasts and Fusobacteria. Fig 4 shows the top 20 genera across all samples. The genus *Escherichia/Shigella* is found in the highest fractions followed by unassigned genus as well as *Propionibacterium*, *Staphylococcus* and *Corynebacterium* in top 5. All samples were tested for the presence of *virA* to rule out some potentially pathogenic *Escherichia* and *Shigella*. No *virA* was detected in the samples. While the use of the *virA* gene fragment has been shown to be a highly specific and sensitive method to detect pathogenic *Shigella* and *Escherichia*[30] it does not allow for the detection of all pathogenic strains within these genera. There is a high degree of variability in the genera found in the different samples, probably reflecting arbitrary input from the environment.

**Fig 5:**
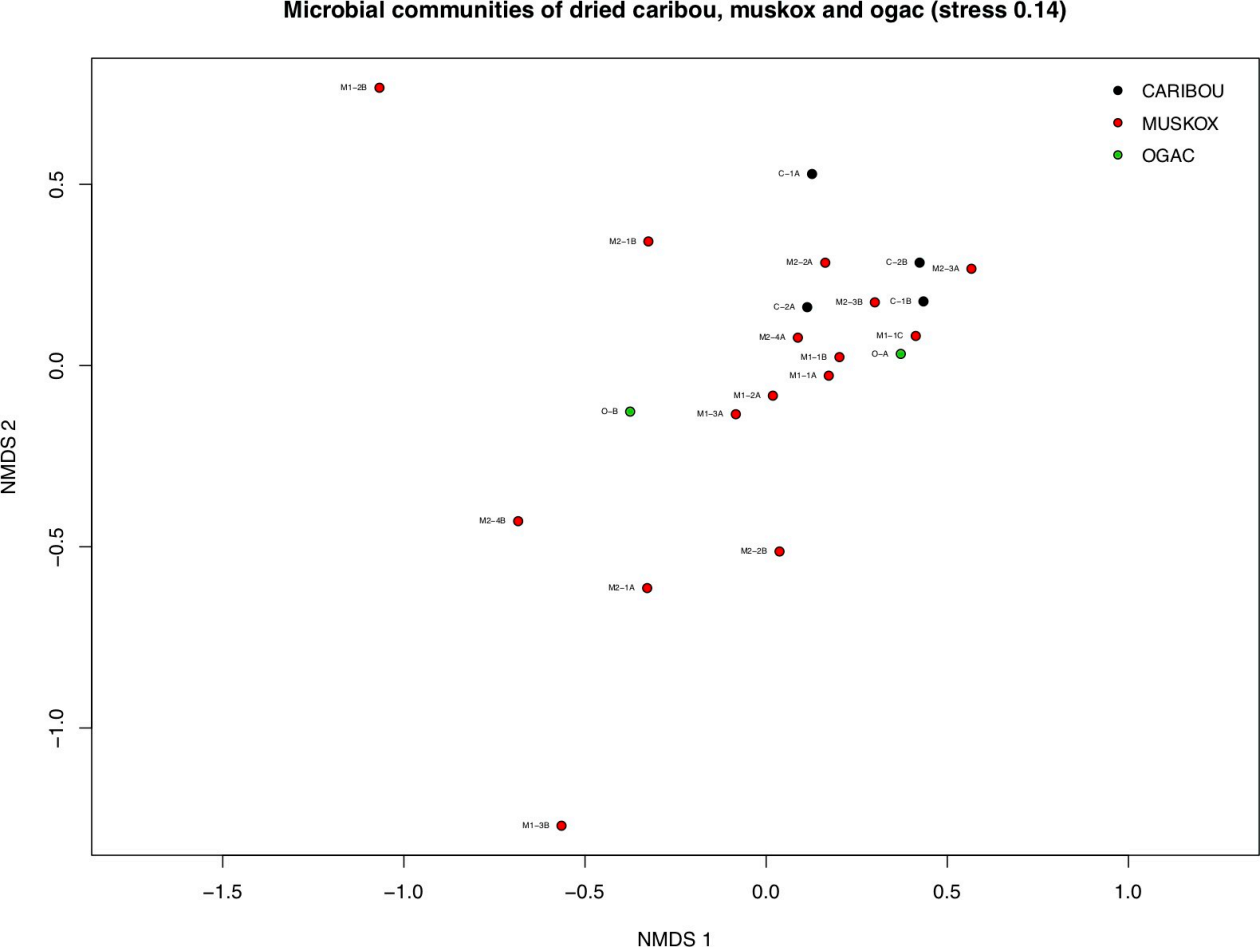
Non-metric multidimensional scaling of samples from dried meat of muskox, caribou and ogac. Please refer to Table 1 for overview of samples and explanation of sample labels.

As seen from Fig 5 the dried samples from all animals do not show clustering based on species of animal (ANOSIM R = -0.137, *p* = 0.737). This suggests that different species of animals do not develop distinct microbial composition while drying in support to the notion that the microbial composition is a result of input from the environment within the timeframe of drying tested in this study. In accordance with this, at the level of individuals, the dried muskox also did not show any separation (ANOSIM R = -0.058, *p* = 0.832).

**Fig 6:**
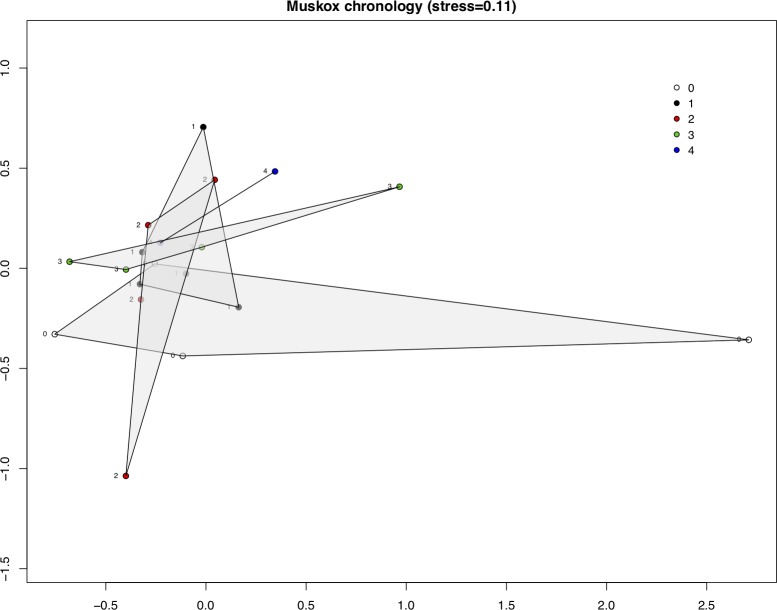
Non-metric multidimensional scaling of muskox samples from muskox 1 and 2 marked with drying time in days. Convex hulls contain samples grouped by drying time in days.

We assessed whether the meat gradually developed distinct microbial compositions during the drying process. The microbial composition on meat from the two muskox individuals did not show distinct clustering of samples from any particular duration of drying (Fig 6, ANOSIM R = -0.021, *p* = 0.563). Samples of fresh muskox (day 0) do show greater variability and a gradually decreasing variability along the duration of drying. While this suggest that the drying process decreases the variability of the microbial community on the dried meat even within the short timeframe studied here, this is not supported by results from caribou where samples from day 0–3 did not at all separate on a two-dimensional NMDS plot (not shown). This leads us to conclude that the comparably fast drying of meat with the objective of limiting microbial growth does not share features of microbial maturation as observed from fermented cured meats.

Previous work has established that the microbes we eat vary in abundance and community composition depending on the foods ingested [5]. The need for a better understanding of the total microbial communities on our foods and how they vary across foods, meals and diets and how food preparation and handling impacts total microbiota of the foods we eat was highlighted [5]. This prior preliminary but seminal work focused on the typical American diet, the USDA recommended diet and a vegan meal plan [5]. The present work contributes to the evolving understanding of food microbiomes by adding an example of a highly un-processed and animal-sourced diet. Our results suggest that animal species and drying process does not select for distinct microbiota and that the resulting microbial composition on dried animal and fish meat assessed in the current study is mostly a result of environmental input to the meat rather than intrinsic properties of the meat itself, at least within the short drying time necessary at the location and climate during this study. These results show how foods are carriers of microbes from the environment in which they are prepared and, at least for naturally prepared foods, that these microbes vary notably among particular pieces of food. Finally, as microbial input from the environment shapes the microbial composition of traditionally dried meat, this encourages the development of hypotheses about how this influence from the environment affects the ecology of our gastrointestinal microbiota when such highly un-processed and naturally prepared products are ingested.

### The caribou rumen is a potential source of microbial byproducts of a plant-rich diet

The caribou rumen has a richness of 441 OTUs (Chao1 442), which is more than twice the highest richness among other samples (Table 1) and in the range of what has been shown for reindeer rumen microbial biodiversity previously [23]. The phyla Bacteroidetes and Firmicutes are dominant in the rumen (Fig 3), in accordance with results from previous studies on Norwegian and Russian reindeer [23,24]. There is a higher fraction of Synergistetes and a lower fraction of Proteobacteria in the rumen compared to other samples. The rumen also has a comparably high fraction of unassigned OTUs at both phylum and genus level when compared to other samples (Figs 3 and 4). The cecum sample has a comparably high fraction of unassigned genera and phyla compared to meat samples and is distinguished by a higher fraction of *Shewanella* than in all other samples (Figs 3 and 4). *Shewanella* are known from a variety of marine environments [39] also including marine animal intestines [40] and marine vegetation [41]. It is tempting to speculate that the caribou might have foraged on marine vegetation, but no marine chloroplasts were detected in the rumen or cecum of the caribou to give any support of such speculation. An alternative explanation could be aerosolization from nearby coastal areas.

The genus *Prevotella* distinguishes the rumen microbiota from all the other microbiota (Fig 4). The rumen microbiota contains 71 different OTUs of *Prevotella*. The presence of a large fraction and diversity of *Prevotella* in a foodstuff of a population that has traditionally eaten very little to no plants encourages certain considerations. *Prevotella* has been shown to be significantly positively correlated with long-term fiber intake [6,9], thus a population like the Inuit eating a diet which would rarely expose the intestinal microbiota to fiber is unlikely to host a microbiota with the necessary genomic potential for fiber-degradation. Such a microbiota would therefore be non-optimal for extracting the benefits from dieting on plants. This is in contrast to indigenous populations dieting on fiber-rich foods, where a large proportion of *Prevotella* is hypothesized to be an adaptation to their fiber-rich diet [9,42]. Therefore, eating the already processed plant-material from herbivores seems an optimal strategy for acquiring healthy byproducts of plant-digestion in a population consuming an extremely low amount of plants. In a previous study the content of n-Butyrate in Norwegian reindeer rumen was measured to be up to 13.4 mg/mL (mean 4.5 mg/mL n = 6) [23]. Coprophagia of herbivore fecal matter could also be part of such a nutrient supplementary strategy and this has been described as part of the traditional Inuit diet in Greenland [43,44]. It has been hypothesized that consuming a diet high in cellulose could also mean consuming a population of microbes well-equipped to digest cellulose [5]. The current study shows that the Inuit have consumed microbes well-equipped to digest plant-material when eating one of the rare plant-based foods in the traditional Greenlandic diet.

In summary, the caribou rumen is a rich source of microbes that has traditionally been ingested and which has previously been shown to contain by-products of microbial fermentation associated with health. Today it is uncommon if not unseen to ingest caribou stomach content in Greenland. Research is encouraged to understand the potential health implications that the ingestion of such might have on a population with a high-protein and high-fat diet. In general, we encourage the expansion of knowledge on rare Inuit foods with the aim of diversifying what can be conceived as local foods of Greenland to help positive developments in food security, food policy and public health.

### Insects and parasites

Maggots of the blue fly are known to spoil drying meat by laying eggs in little folds on drying caribou meat [20]. At the drying location blow-flies were abundant and left visible traces in the form of excreta and maggots (Fig 1D and 1E). While maggots are sought to be avoided when preparing the meat, e.g. by limiting folds in the meat pieces, they are an unavoidable part of the drying process and they are not considered harmful. Besides maggots another parasite was observed in the meat (Fig 1F and 1G). Based on photographs these were evaluated to being the *Taenia* cf. *krabbei*, a type of helminth first described in muskox from southwest Greenland in 2012 [45]. *Taenia* cf. *krabbei* has a lifecycle step in the intestines of carnivores before excretion and transfer through vegetation to a herbivorous intermediate host such as the muskox and caribou. Here the larva form cysts in the host organs and skeletal muscle. This parasite has not been reported as zoonotic and cannot establish itself in a human host [45,46].

The microbiota of the Oestridae parasitic larvae of the caribou hide has an intermediate OTU richness of 143 compared to other samples (Table 1). These OTUs are comprised of a large fraction of unassigned phyla as well as the phyla Proteobacteria, Actinobacteria, Firmicutes and some Bacteroidetes (Fig 3). The dominant genera found in the larvae microbiota are *Escherichia/Shigella*, *Propionibacterium*, smaller fractions of *Staphylococcus*, *Corynebacterium*, *Kocuria*, *Streptococcus*, *Acinetobacter* and *Lactococcus* and finally a large fraction of OTUs unassigned at genus level (Fig 4). There are 17 OTUs unique to the larvae sample, showing that the larvae are likely to add to the microbial diversity ingested in connection to hunting activities assessed in the current study. It can be assumed that the microbes found on the larvae originate either from the larval microbiota or the environment. Among the less common taxa of the exclusive larva OTUs is the genus *Elizabethkingia*, that has been isolated from mosquitoes among other places [47] as well as *Asticcacaulis*, previously isolated from tundra wetland soil [48] in support of the above assumption.

## Conclusions

It can be concluded that natural drying of meat using traditional methods of the Inuit is efficient in controlling microbial spoilage, in accordance with local knowledge. Animal species and drying processes do not select for distinct microbiota and the resulting microbial composition on dried meat assessed in the current study is concluded to be a result of environmental input, emphasizing the high variability of microbes on foods and the importance of food origin. The biodiversity and microbial composition of foods stemming from traditional hunting activities in Greenland shows the potential that these foods have as carriers of microbes from the environment to the human gastrointestinal tract. The caribou rumen is a rich source of microbes that has traditionally been ingested and which has previously been shown to contain by-products of microbial fermentation that have been associated with positive health impact. The established presence of a number of edible parasites represent a diversity of food in themselves that have been lost from the modern diet in Greenland, as parasites are now sought to be prevented rather than seen as a potential source of nutrients. This work acts as an initial step towards understanding the microbial dynamics of the nature-sourced and gradually disappearing traditional Inuit diet as well as a reference point for a better understanding in future studies into the gut microbiome of the Inuit.

## Ethical statement

The animals sampled in this study were hunted for consumption as part of local hunting activities and were not killed for the purpose of research. This research was conducted with prior informed consent from the Government of Greenland survey license G17-025.
